# Cystic Renal Masses: From Malignancy Probability to Risk-Adapted Management

**DOI:** 10.3390/cancers18193095

**Published:** 2026-09-23

**Authors:** Chencan Fang, Chenyi Ge, Lijuan Yin, Xin Lu, Le Qu, Hanzhong Zhang, Yan Zhang, Ying Liu, Yu Fang, Qinqin Kang, Aimin Jiang, Linhui Wang

**Affiliations:** 1 Department of Urology, Changhai Hospital, Naval Medical University, Shanghai 200433, China; chencanfang2003@163.com (C.F.); gechenyi666@163.com (C.G.); luxin_smmu@163.com (X.L.); zhz1930854233@163.com (H.Z.); 18905232105@163.com (Y.Z.); liuying03240@163.com (Y.L.); 13916943129@163.com (Y.F.); 2 Department of Pathology, Changhai Hospital, Naval Medical University, Shanghai 200433, China; yinlijuan2@163.com; 3 Department of Urology, Affiliated Jinling Hospital, Medical School of Nanjing University, Nanjing 210002, China; septsoul@smmu.edu.cn; 4 Department of Radiology, Changhai Hospital, Naval Medical University, Shanghai 200433, China; kangqinqin@live.cn

**Keywords:** cystic renal masses, Bosniak classification, renal cell carcinoma, biological aggressiveness, active surveillance, partial nephrectomy

## Abstract

Cystic renal masses (CRMs) encompass a broad spectrum of conditions ranging from simple renal cysts to cystic renal cell carcinoma. Their detection has increased markedly with the widespread use of abdominal imaging, raising concern about potential overtreatment: many complex CRMs treated surgically are ultimately benign, while some malignant lesions are low grade, localized, and follow a relatively indolent clinical course. Although the Bosniak classification stratifies malignancy probability, translating lesion characteristics into preoperative assessments of biological aggressiveness and patient-specific clinical consequences remains challenging. Drawing on published evidence and common clinical decision-making scenarios, this Perspective outlines the pathologic classification of CRMs, examines the limitations of the Bosniak classification, and discusses the prospects for and current barriers to the use of radiomics and artificial intelligence for benign-versus-malignant classification. With the dual objectives of differentiating benign from malignant lesions and assessing biological aggressiveness, we focus on individualized management strategies that integrate the probability of malignancy, potential clinical harm, and patient-specific factors. These strategies include conservative management, indications for surgery, and selection of surgical approach. The aim is to inform more standardized and precise diagnosis and management of CRMs.

## Simple Summary

Cystic renal masses range from simple benign cysts to kidney cancers with cystic features. They are being detected more often as abdominal imaging becomes increasingly common. Although some complex cystic masses require treatment, many surgically removed lesions are benign, and some malignant lesions are low-grade, confined to the kidney, and slow-growing. Current assessment focuses mainly on whether a mass is likely to be malignant, whereas how aggressively it may behave and the harm it may cause are more difficult to estimate before treatment. This Perspective reviews the range of conditions that can present as cystic renal masses, the role and limitations of the Bosniak imaging classification, and emerging applications of computer-based image analysis and artificial intelligence. It also discusses how malignancy risk, potential aggressiveness, patient health, kidney function, and individual preferences may be considered together when selecting surveillance or intervention.

## 1. Introduction

Cystic renal masses (CRMs) in adults encompass a broad spectrum of focal lesions, ranging from simple renal cysts (SRCs) to cystic renal neoplasms. With the routine use of abdominal imaging in health screening, the detection of CRMs has increased markedly; approximately half of individuals aged 50 years or older have at least one renal cyst [1]. This increase has been accompanied by concerns about overtreatment. A proportion of Bosniak III lesions are still found to be benign after surgery, whereas Bosniak IV lesions generally have a higher likelihood of malignancy [2]. However, a higher likelihood of malignancy does not necessarily indicate greater biological aggressiveness or clinical harm. Some low-grade, kidney-confined renal cell carcinomas (RCCs) composed predominantly of cystic components follow a relatively indolent clinical course, suggesting that the intensity of treatment may exceed the actual oncologic risk in some patients. Although the Bosniak classification primarily estimates malignancy probability, current management guidance also incorporates patient characteristics and treatment-related risks [3]. Building on the established distinction between identifying malignancy and identifying aggressive disease [4], this Perspective provides a CRM-focused synthesis of histologic heterogeneity, preoperative assessment, and management. Its contribution is to distinguish findings that raise concern for aggressive disease from factors that inform patient-specific clinical consequences, and to link these assessments to surveillance, intervention, and longitudinal reassessment.

## 2. Classification of Cystic Renal Lesions

Terminology for renal neoplasms in this section follows the fifth edition of the World Health Organization (WHO) Classification of Tumours: Urinary and Male Genital Tumours (2022) [5]. Cystic appearance is an imaging or gross morphologic feature shared by several renal tumor entities and does not constitute a single histologic diagnosis. Diffuse inherited cystic disorders such as polycystic kidney disease require a different assessment approach from sporadic focal CRMs [6].

### 2.1. Benign Lesions and Background Cystic Conditions

#### 2.1.1. Simple Renal Cysts

SRCs are the most common cystic renal lesions in adults. They may be solitary or multiple and range from a few millimeters to several centimeters in diameter. The cyst wall is lined by a single layer of epithelium. On ultrasonography, SRCs appear as round or oval anechoic lesions with thin, smooth walls and posterior acoustic enhancement; on abdominal computed tomography (CT), they show homogeneous fluid attenuation without enhancement [7]. SRCs are generally considered acquired changes associated with aging. Early microanatomic studies suggested that they may be related to diverticulum-like dilation of the distal renal tubules or collecting ducts [8]. Observational studies have also suggested possible associations among SRCs, nephron loss, compensatory changes in the remaining nephrons, and activity of the renin–angiotensin–aldosterone system, although the underlying mechanisms and causal relationships remain incompletely defined [7,9]. Most patients are asymptomatic, and the cysts are discovered incidentally during health examinations; a minority develop flank pain, hematuria, or abdominal discomfort because of cyst enlargement, hemorrhage, infection, or local compression. SRCs are epidemiologically associated with hypertension, but the causal relationship remains debated [10].

#### 2.1.2. Parapelvic Cysts

Parapelvic cysts typically arise from the renal parenchyma adjacent to the renal sinus and protrude into the sinus; pathologically, they generally resemble SRCs [11]. Owing to their distinctive anatomic location, they are more likely than SRCs to cause clinical symptoms. In a multicenter retrospective clinical cohort, Wang et al. [12] reported that 77.85% of the 158 patients with parapelvic cysts were symptomatic and 72.15% had compression of the collecting system; hydronephrosis was recorded in 29 patients. Some patients may also have hypertension, possibly because local compression reduces renal parenchymal perfusion and activates the renin–angiotensin–aldosterone system through a mechanism analogous to that of Page kidney. Parapelvic cysts may provide a clue to Fabry disease when accompanied by suggestive extrarenal findings or family history [13]. On imaging, parapelvic cysts should also be distinguished from hydronephrosis and peripelvic cysts. If surgery is required, their anatomic relationships with the renal vessels and collecting system should be delineated preoperatively.

#### 2.1.3. Peripelvic Cysts

Peripelvic cysts are generally considered to arise from dilated lymphatic channels within the renal sinus and are lined by lymphatic endothelium. Their development has been associated with congenital embryonic lymphatic remnants or acquired obstruction of lymphatic drainage. These lesions are often multiple and bilateral and appear as confluent, irregularly shaped, thin-walled anechoic lesions in the renal sinus. During the excretory phase of CT, contrast material does not enter the cyst cavity, which helps distinguish these lesions from hydronephrosis [14,15]. Because they are generally small and distributed around the collecting system, most peripelvic cysts are asymptomatic and do not require treatment [14].

#### 2.1.4. Calyceal Diverticulum

A calyceal diverticulum (CD) is a nonsecretory outpouching of the upper urinary collecting system located within the renal parenchyma. It is lined by urothelium, communicates with the collecting system through a narrow neck, and passively fills with urine [16]. The precise etiology of CDs remains unclear. Most studies favor a congenital origin, possibly related to abnormal formation of the ureteric bud; a minority may develop secondary to infection, obstruction, trauma, or vesicoureteral reflux [17]. Because of their narrow neck and lack of active drainage, CDs can be misidentified as SRCs on ultrasonography or CT. Gradual filling with contrast material on delayed or excretory-phase contrast-enhanced CT establishes the diagnosis [18]. Most patients are asymptomatic, and the lesion is detected incidentally on imaging; symptomatic patients most commonly present with ipsilateral flank pain, recurrent urinary tract infections, or hematuria [16,17]. Calculi are the most common complication, with a reported prevalence of 9.5–50% [16], and are thought to result from the combined effects of urinary stasis and metabolic abnormalities such as hypercalciuria [19]. In addition, urothelial carcinoma or squamous cell carcinoma arising within a CD has been described in a very small number of case reports [17].

#### 2.1.5. Adult Cystic Nephroma

Adult cystic nephroma (ACN) is a rare benign multilocular cystic renal tumor [20,21]. ACN and mixed epithelial and stromal tumor (MEST) lie along an overlapping morphologic spectrum: ACN is predominantly cystic, whereas MEST usually contains a larger solid stromal component, and definitive classification requires complete histologic assessment. Both predominantly affect middle-aged, perimenopausal women and have been reported in association with exogenous hormone exposure or hormone receptor expression, although a direct causal relationship has not been established. Histologically, they show a biphasic architecture in which the epithelium forms cysts or tubules, often with hobnail cells, and the stroma shows spindle-cell proliferation with “ovarian-like” features [20,21]. Most patients with ACN are asymptomatic; symptomatic patients most commonly present with abdominal pain, hematuria, or an abdominal mass [20]. On CT or magnetic resonance imaging (MRI), these lesions usually appear as multilocular cystic masses. They are often classified as Bosniak III because of thickening or enhancement of the cyst wall or septa, and those with nodular enhancement may be classified as Bosniak IV; preoperative distinction from cystic RCC remains difficult [20].

#### 2.1.6. Acquired Cystic Kidney Disease

Acquired cystic kidney disease (ACKD) refers to acquired cystic changes in the kidneys of patients with end-stage renal disease (ESRD) who do not have hereditary polycystic kidney disease. ACKD can develop before dialysis, and its prevalence increases with dialysis duration, suggesting that it is primarily associated with longstanding kidney failure rather than dialysis itself. Pathologic and molecular studies suggest that ACKD cysts may originate from the renal tubular epithelium and involve alterations in MET/HGF signaling and other pathways. Atypical cysts are considered possible precursor lesions of acquired cystic disease-associated renal cell carcinoma (ACD-RCC) [22,23]. Most patients are asymptomatic; a minority develop flank pain and hematuria because of intracystic hemorrhage. The risk of RCC is substantially increased in patients with ACKD. ACD-RCC is a common RCC subtype in the setting of ESRD and ACKD and may be multifocal or bilateral and may metastasize [22,24].

#### 2.1.7. Other Rare Benign Cystic Lesions

Other rare benign lesions include renal lymphangiomas and hydatid cysts. Although uncommon in clinical practice, they should not be overlooked in the differential diagnosis. Renal lymphangioma is a developmental malformation of the renal lymphatic system; impaired lymphatic drainage can result in thin-walled cystic dilations in the perirenal space, renal sinus, or renal parenchyma. Most patients are asymptomatic, whereas some develop flank pain, hematuria, or hypertension because of cyst compression. Extensive or bilateral lesions, or those causing substantial compression, may impair kidney function [25]. Renal hydatid cysts account for approximately 4% of all cases of hydatid disease. Their imaging appearance may overlap with that of complex cysts or cystic tumors; wall calcification and the daughter-cyst sign are characteristic findings, and the diagnosis should be considered in pastoral areas. Patients most commonly present with flank pain. Communication between the cyst and the collecting system may cause characteristic hydatiduria, whereas rupture can lead to a severe anaphylactic reaction [26]. A history of epidemiologic exposure and characteristic imaging findings may help prevent misdiagnosis as a cystic tumor and the resulting unnecessary surgery.

### 2.2. Neoplasms of Low Malignant Potential or Borderline Lesions

Because of its indolent biological behavior, the 2012 International Society of Urological Pathology Vancouver Consensus recommended renaming multilocular cystic renal cell carcinoma as multilocular cystic renal neoplasm of low malignant potential (MCRNLMP) [27]; this designation was formally adopted in the 2016 WHO classification. MCRNLMP accounts for <1% of all renal tumors and is composed entirely of multiple cysts. The septa contain small aggregates of low-grade clear cells, without expansile growth, mural nodules, or necrosis. Most lesions are classified as Bosniak IIF or III. A minority may be classified as Bosniak IV because of nodular enhancing areas or a pseudosolid appearance created by converging septa. Preoperative distinction from low-grade clear cell renal cell carcinoma (ccRCC) with a predominant cystic component remains difficult, and definitive diagnosis requires comprehensive histopathologic sampling of the resection specimen [28]. Chromosome 3p deletion and VHL alterations support a molecular relationship between MCRNLMP and ccRCC [29,30]. However, these shared alterations are not sufficient to distinguish the two entities; histologic growth pattern and cytologic features, particularly the presence or absence of expansile solid growth, remain central to their distinction [28,29]. MCRNLMP predominantly occurs in middle-aged adults, with a slight male predominance. Most patients are asymptomatic and the lesion is discovered incidentally during health examinations; a minority present with flank pain or hematuria [28].

### 2.3. Malignant Lesions

A distinction should be made between tumors that are truly composed predominantly of cystic components and solid RCCs with pseudocystic change caused by hemorrhage or necrosis, as their biological behavior differs. Among cystic RCCs, ccRCC with extensive cystic change (cystic ccRCC) is the most common form, with expansile nests of clear cells within fibrous septa. In a cohort of low-grade cystic renal epithelial tumors that underwent centralized pathologic review, the 10- and 20-year cancer-specific survival rates were both 100%; however, this finding cannot be extrapolated to all cystic ccRCCs [31]. From a preoperative imaging perspective, high-risk features include enhancing mural nodules or a substantial solid component, which raise suspicion of malignancy, and venous invasion or extrarenal extension, which suggest invasive disease; the definitive pathologic subtype and grade still require histologic examination. Tissue morphology can also reflect underlying vascular, epithelial, inflammatory, and microenvironmental processes that may contribute to organ dysfunction and clinical manifestations [32]. Accordingly, pathological findings should be interpreted alongside imaging characteristics and clinical context to inform risk-adapted management. Among other subtypes, a pathologically confirmed cohort showed that approximately 17% of papillary renal cell carcinomas (pRCCs) had a cystic appearance [33]. Microscopically, a microcystic architecture at the tumor–stroma interface was associated with extrarenal invasion and metastasis [34]; however, this postoperative histologic feature is not equivalent to a cystic appearance on imaging and cannot be used directly for preoperative risk stratification. Tubulocystic RCC is recognized as a distinct entity in the 2022 WHO classification. It is composed of cystic tubules and dilated ducts lined by eosinophilic epithelium. Most pure cases follow a relatively indolent clinical course, although a small number of metastatic cases have been reported [35,36]. As discussed above, ACD-RCC occurs primarily in the setting of ESRD and ACKD and is heterogeneous in pathologic grade and clinical behavior; it should not be grouped with MCRNLMP or low-grade cystic ccRCC and uniformly regarded as indolent. Table 1 summarizes selected histologic entities discussed in this section, highlighting their key features and implications for diagnosis and evidence interpretation.

## 3. Major Diagnostic Challenges

### 3.1. Limitations of the Bosniak Classification

Successive revisions of the Bosniak classification have sought to improve the reproducibility and specificity of malignancy risk stratification for CRMs. The system primarily estimates the likelihood of malignancy rather than directly predicting tumor grade, metastatic potential, or patient outcomes. Overall interobserver agreement is similar between the 2019 and 2005 versions, with wall and septal morphology remaining the main sources of disagreement [37]. A meta-analysis comprising pathologically confirmed cases reported malignancy rates of 79% and 84% for Bosniak v2019 class III and IV lesions, respectively [38]; these estimates are affected by surgical selection and pathologic verification bias and should not be interpreted as indicating that the two categories are clinically equivalent. Existing pooled studies have not demonstrated significant differences in malignancy rates among the imaging subtypes of class III lesions [39]. This uncertainty in the gray zone directly affects clinical decision-making: up-classification may increase unnecessary surgery, whereas conservative surveillance may miss lesions that truly progress. In our view, morphologic thresholds inevitably have limits, and measurement differences of 1–2 mm are difficult to avoid given current imaging resolution. When classification is disputed, multidisciplinary review combined with additional imaging and longitudinal follow-up is more informative than rigid reliance on a single classification result.

### 3.2. Radiomics and Artificial Intelligence: Opportunities and Challenges

When standardized renal contrast-enhanced CT or MRI cannot adequately characterize a lesion, contrast-enhanced ultrasound (CEUS) may serve as an adjunct in selected patients; however, no standardized sequential multimodality imaging pathway is currently applicable to all CRMs [40]. Some studies have reported that the sensitivity of CEUS for malignant CRMs approaches 100%, although its relatively low specificity may also lead to more benign lesions being referred for surgery or intensive surveillance [41]. In a retrospective series of resected lesions, Herms et al. [42] found that CEUS assigned 21 of 52 lesions (40.4%) to a higher Bosniak category than CT/MRI. Among histologically verified CEUS-Bosniak class III lesions, Járay et al. [43] reported a positive predictive value for malignancy of 25% (4/16), an estimate potentially affected by verification bias. Clinicians should therefore remain cautious. Up-classification resulting from greater imaging sensitivity is not equivalent to malignant progression; using CEUS-based up-classification alone to recommend surgery may exacerbate overtreatment.

Radiomics and artificial intelligence (AI) offer quantitative approaches to CRM characterization. Miskin et al. [44] analyzed 144 CRMs using single-slice contrast-enhanced CT texture features; three classifiers achieved areas under the receiver operating characteristic curve (AUCs) of 0.76–0.80 with internal tenfold cross-validation. Huang et al. [45] used manual whole-lesion segmentation for unenhanced CT radiomics in 207 pathologically confirmed CRMs, with 162 training lesions and 45 external validation lesions from a second center. Their k-nearest neighbor model achieved an external AUC of 0.86 (95% CI, 0.74–0.98). He et al. [46] combined interactive three-dimensional segmentation with a multiphase CT deep-learning classifier. The classification cohorts comprised 195 training lesions, 83 validation lesions, and 162 test lesions pooled from three external centers; the external test AUC was 0.998 (95% CI, 0.993–1.000).

Huang et al. [45] reported calibration curves showing some deviation from ideal calibration in the external cohort. These studies evaluated benign-versus-malignant classification rather than progression, metastasis, or cancer-specific mortality [44,45,46]. Moving beyond benign–malignant classification, Kang et al. [47] studied 1138 patients with pathologically confirmed CRMs and developed a simplified CT model using manually assessed septal number and convex protrusion angle. Its predicted malignancy probability was then combined with clinical and lesion characteristics in an extreme gradient boosting (XGBoost) model to distinguish study-defined indolent from aggressive lesions. The XGBoost model achieved AUCs of 0.92 and 0.80 in the internal validation and first external test cohorts, respectively. Model evaluation restricted to histologically confirmed lesions may be affected by selection and verification bias. When pathology is available, studies should report cohort histologic subtypes, grades where applicable, and the labeling of MCRNLMP.

In our view, near-term AI applications include assisting segmentation, standardizing assessment, and providing a second opinion on malignancy probability. Clinical utility cannot be inferred from retrospective diagnostic performance alone; routine implementation requires external validation, generalizability across patient populations, interpretability, workflow integration, and prospective evidence of clinical benefit [48]. A future priority is to establish a standardized multicenter CRM database in China linked to longitudinal outcomes.

### 3.3. Dual Diagnostic Goals: Differentiating Benign from Malignant Lesions and Assessing Biological Aggressiveness

The two diagnostic goals of estimating malignancy probability and assessing biological aggressiveness should be distinguished from the separate assessment of patient-specific clinical consequences. Silverman et al. [4] have previously articulated the distinction between identifying malignancy and identifying aggressive disease. This Perspective applies that distinction to CRMs by relating available preoperative findings to patient-specific clinical consequences and management decisions. Here, biological aggressiveness denotes invasive and metastatic potential; high-risk features are lesion-level findings suggesting malignancy or aggressive behavior; and potential clinical harm encompasses adverse consequences of the lesion or its management. In practice, reliable prediction of future aggressive behavior remains difficult in localized CRMs without overt invasion. In a retrospective cohort of 387 radiographically cystic renal lesions, Lee et al. found that Bosniak class was associated with high-grade pathology in the intervention subgroup [49]. This finding supports considering Bosniak class when assessing the likelihood of high-grade pathology, while distinguishing this histologic association from prediction of future progression or metastasis. The models summarized in Section 3.2 address malignancy classification and exploratory aggressiveness assessment; evidence for the molecular markers discussed above is mainly derived from postoperative tissue studies. Renal mass biopsy likewise has limited diagnostic performance: the nondiagnostic rate for cystic lesions may be as high as 39.8%, whereas the reported 62.5% concordance rate for four-tier nuclear grading was derived from data on renal masses in general rather than evidence specific to cystic lesions; intratumoral heterogeneity can also lead to grade underestimation [50]. Accordingly, the Canadian Urological Association (CUA) guideline suggests considering biopsy for Bosniak IV CRMs with a substantial solid component amenable to safe sampling when the result could alter management [3]. Luomala et al. [51] retrospectively studied 532 patients with Bosniak IIF–IV CRMs evaluated during 2006–2017, before publication of Bosniak v2019, and found no significant difference in 5-year metastasis-free survival between active surveillance (AS) and initial surgery. However, 85 of 106 patients with Bosniak IV lesions underwent initial surgery, and treatment selection was based on clinician and patient preferences rather than a standardized protocol. This selection limits the comparability of treatment groups and the generalizability of AS outcomes to unselected Bosniak IV lesions. The 2023 CUA guideline supports AS for selected small Bosniak III/IV lesions; guideline-specific size criteria and evidence certainty are discussed in Section 4.2 and Section 4.3 [3]. Patient-specific clinical consequences may diverge from biological aggressiveness: a symptomatic benign cyst may cause clinically meaningful morbidity, limited life expectancy may reduce the expected benefit of intervention, and a solitary kidney may increase the functional consequences of treatment, without any of these factors indicating greater biological aggressiveness [3,52]. Figure 1 summarizes the complementary assessment of malignancy probability and concern for biological aggressiveness.

### 3.4. Pitfalls and Confounders in Differential Diagnosis

Hemorrhage, infection, and other secondary changes can complicate the imaging appearance of CRMs, while some lesions outside this category may also have a cystic appearance. Intracystic hemorrhage may increase cyst attenuation and lead to a higher Bosniak category; although MRI can identify T1 hyperintensity caused by subacute hemorrhage, some pRCCs may also be T1 hyperintense [53,54], and postcontrast subtraction imaging can be used to determine whether true enhancement is present [1]. Intracystic infection may manifest as wall thickening, intracystic debris or gas, and surrounding fat stranding, with features that overlap those of cystic RCC; much of the relevant evidence derives from patients with autosomal dominant polycystic kidney disease (ADPKD) [54]. When extrapolating these findings to sporadic cystic lesions, fever, inflammatory markers, and changes after treatment should be considered together. When infection or abscess is strongly suspected clinically, image-guided aspiration may be performed for microbiologic diagnosis and drainage; when the neoplastic nature of the lesion remains uncertain, aspiration of cyst fluid alone should not be used routinely for differential diagnosis. According to Bosniak v2019, calcification itself is not an independent sign of malignancy, although coarse calcification may obscure adjacent enhancing tissue; MRI with subtraction imaging can be used when necessary [55]. The position-dependent calcium–fluid layering seen with renal milk of calcium should be differentiated from calculi to avoid inappropriate lithotripsy [56]. Rare cases of urothelial carcinoma arising within a CD have also been reported and may present as a cystic lesion that communicates with the collecting system and contains a soft-tissue filling defect or papillary component [57]. Invasive upper tract urothelial carcinoma typically demonstrates hypoenhancement, an infiltrative growth pattern, and relative preservation of the renal contour, and generally lacks the characteristic hypervascular enhancement of ccRCC [58]. When biopsy or surgical tissue is available, immunohistochemical markers such as GATA3, p63, and PAX8 can assist pathologic differentiation, but they must be interpreted together with tissue morphology and the full immunophenotypic profile [59,60]. Diagnostic findings should also be integrated with prognostic information and clinical context to support individualized risk assessment, particularly when diagnostic uncertainty influences the choice between surveillance and intervention [61].

## 4. Management Strategies

### 4.1. Risk of Overtreatment

The risk of overtreatment in CRMs extends beyond unnecessary intervention for benign lesions: even for malignant lesions, the timing and intensity of treatment should be proportionate to the expected clinical benefit. When both intervention and surveillance are reasonable options, the question is whether immediate treatment offers a meaningful advantage over surveillance with selective delayed intervention. In a cohort of 387 CRMs, Lee et al. [49] reported that 247 underwent intervention and that 23% of the treated lesions showed high-grade histology; Lucocq et al. [2] reported that 20.7% of surgically treated Bosniak III lesions classified using pre-v2019 criteria were benign. Resection of benign lesions highlights an opportunity to improve preoperative selection, whereas the presence of high-grade tumors underscores the importance of identifying lesions likely to benefit from intervention [2,49]. In a series of 76 patients with MCRNLMP, 66 were followed for a median of 52 months after surgery, with no recurrence or metastasis [62]. This favorable postoperative course is consistent with the indolent behavior of histologically confirmed MCRNLMP. In Lee et al.’s AS cohort, the linear growth rates of Bosniak IIF, III, and IV lesions were 0.6, 0.5, and 2.7 mm/year, respectively, and growth rate was not significantly associated with high-grade histology [49]. In Luomala et al.’s cohort, 15 of 21 patients with Bosniak IV lesions initially managed with AS subsequently underwent surgery [51]. Deferring surgery and avoiding surgery altogether are therefore distinct outcomes; the timing and oncologic outcomes of subsequent intervention matter when assessing whether surveillance reduces overtreatment. Treatment-related burdens, including perioperative morbidity and loss of renal function after partial nephrectomy (PN), should be weighed against the expected oncologic benefit of immediate intervention [52]. Accordingly, risk-adapted management should aim not merely to reduce intervention, but to match treatment intensity and timing to the expected oncologic benefit—deferring treatment when the immediate benefit is limited while preserving the opportunity for timely curative intervention if high-risk features emerge or progress.

### 4.2. Observation and Active Surveillance

Bosniak I and II lesions carry an extremely low risk of malignancy; asymptomatic lesions that can be confidently classified require neither treatment nor routine follow-up. For Bosniak IIF lesions, follow-up is recommended at 6 and 12 months during the first year and annually thereafter if stable, for a total follow-up period of 5 years [63,64]. In selected populations at increased risk of RCC, such as kidney transplant recipients with ACKD, targeted RCC screening has been proposed [65]. The 2023 CUA guideline recommends Bosniak v2019, favors AS for class III/IV lesions ≤2 cm, and allows AS or surgery for lesions measuring 2–4 cm; these are conditional recommendations supported by low- and very-low-certainty evidence, respectively [3]. In contrast, the 2026 National Institute for Health and Care Excellence (NICE) guideline frames its management recommendations by Bosniak category and lesion size, advising consideration of AS for Bosniak III/IV lesions <2 cm and for Bosniak III lesions ≥2 cm [66]. AS entails scheduled imaging surveillance with delayed intervention when the risk changes, whereas watchful waiting is intended for patients in whom competing mortality risk predominates and symptom control is the main goal; the two strategies should not be conflated. The implementation of AS nevertheless faces practical challenges, including patients’ concerns about tumor progression, clinicians’ concerns about missed diagnoses or delayed treatment, and poor adherence to long-term follow-up. Multidisciplinary shared decision-making, standardized follow-up pathways, and digital platforms that support longitudinal follow-up and imaging data management, together with clear communication of the potential benefits, risks, and uncertainties of AS, may improve its feasibility.

### 4.3. Indications for and Timing of Surgery

Indications for surgery should take into account malignant potential, concern for biological aggressiveness, clinical symptoms, and diagnostic uncertainty. Bosniak III/IV lesions that show progressive growth, morphologic or stage progression, associated symptoms, or a change in patient preference should be reassessed and intervention discussed. A sustained growth rate of >0.5 cm/year may serve as one trigger for transitioning to treatment but should not automatically be equated with an indication for surgery. For Bosniak III/IV lesions >4 cm, the 2023 CUA guideline conditionally favors surgery on the basis of low-certainty evidence [3]. The 2026 NICE guideline recommends offering surgery for Bosniak IV lesions ≥2 cm when surgery is suitable; if surgery is unsuitable or declined, AS is one of the alternative options that may be considered [66]. Thus, for Bosniak IV lesions measuring 2–4 cm, CUA allows either AS or surgery, whereas NICE generally favors surgery. This disagreement should be explicitly discussed during shared decision-making [3,66]. Intractable pain, recurrent hematuria or infection, and compression-related symptoms can also occur in benign cystic lesions and may provide a basis for intervention [67]. When imaging cannot reliably distinguish benign from malignant disease, biopsy is infeasible or unlikely to yield a reliable result, and definitive pathologic characterization would materially affect treatment decisions, surgery remains a reasonable option for obtaining a complete pathologic assessment. In patients with a solitary kidney, bilateral lesions, or impaired kidney function, tumor control should be balanced against preservation of kidney function, with nephron-sparing strategies prioritized when technically and oncologically feasible [52]. With regard to timing, in our view, lesions that are highly suspicious for malignancy and progressing rapidly should be managed promptly. When intracystic infection is present or bleeding is difficult to control, drainage, hemostasis, or surgery should be selected according to the clinical circumstances. Malignant or suspected malignant lesions assessed as having a low risk of progression, as well as symptomatic benign cysts, may be managed electively.

### 4.4. Selection of Surgical Approach

When surgery is chosen for Bosniak III/IV CRMs, the CUA guideline suggests PN over radical nephrectomy (RN) when technically and oncologically feasible [3]. The American Urological Association guideline recommends prioritizing PN for cT1a renal tumors that require intervention, whereas the European Association of Urology guideline recommends PN for T1 tumors; in appropriately selected localized renal tumors, PN provides oncologic control comparable to RN while better preserving renal function [52,68]. During robot-assisted partial nephrectomy (RAPN), near-infrared fluorescence (NIRF) assists visualization of renal vasculature and assessment of renal perfusion [69]. In a general renal tumor series, NIRF was used to distinguish exposed tumor from normal renal parenchyma, with a positive surgical margin rate of 0.3% [70]. For completely endophytic tumors, intraoperative ultrasonography aids localization, whereas NIRF-based tumor visualization is limited when the tumor is not exposed [70,71]. For these tumors, enucleation was associated with better renal functional preservation than standard resection [72]. A retrospective cohort of 85 cystic RCCs also reported favorable oncologic outcomes with off-clamp RAPN using enucleation or enucleoresection [73]. Accordingly, enucleation may be considered for CRMs when an appropriate dissection plane permits complete and intact removal.

Pradere et al. [74] reported intraoperative cyst rupture in 50 of 268 patients (18.7%), with no local or metastatic recurrence in the rupture subgroup; median follow-up for the overall cohort was 32 months. In contrast, a subsequent single-center study of 406 patients undergoing PN for pathologically confirmed malignant CRMs reported recurrence in 4 of 32 patients with rupture and 5 of 374 without rupture [75]. On the basis of oncologic surgical principles, we advocate intact resection with every effort to avoid cyst fluid spillage. The RUPTURE Delphi consensus supports prompt suction of spilled tumor tissue, whereas consensus was not reached on irrigation [76]. As expert-opinion-based technical measures for cyst rupture, we suggest prompt suction of spilled fluid, selective irrigation, and closure of the renal resection defect after tumor removal. In a single-center series of 83 patients, preserving overlying adipose tissue during manipulation of exophytic cystic lesions and placing the resection line approximately 0.5 cm from the cyst wall were recommended [77]. Autologous perinephric fat has been used to reconstruct renal defects after tumor excision or enucleation in open PN [78]; accordingly, we suggest using a perinephric fat pad to fill the residual cavity after enucleation of a CRM to reduce local tension and facilitate closure. In a general PN cohort, tumor-bed frozen sections were positive in only 9 of 30 cases with positive final specimen margins [79]; therefore, they cannot replace permanent-section assessment of the resection specimen.

RN may be considered when PN is not technically feasible or would not provide adequate oncologic control [52,68]. Evidence from renal cancer in general suggests that robot-assisted RN was associated with fewer perioperative complications and shorter hospital stay compared with open surgery; however, it offers no clear advantage over laparoscopic surgery and is more costly [80]. Table 2 summarizes the evidence basis and clinical applicability of the diagnostic tools and management measures discussed throughout this Perspective, and Figure 2 illustrates the overall risk-adapted management framework. Within this framework, lesion-level assessment should be integrated with patient-specific clinical consequences, treatment-related considerations, diagnostic uncertainty, shared decision-making, and longitudinal reassessment.

## 5. Conclusions

Standardized diagnosis and management of CRMs should extend beyond differentiation between benign and malignant lesions to include assessment of biological aggressiveness and potential clinical harm. The Bosniak classification provides a practical framework for stratifying the probability of malignancy, but its limited interobserver agreement and inability to directly predict histologic grade, progression, or metastasis make it insufficient on its own to support precise clinical decision-making. In our view, low-risk lesions may be managed with follow-up or AS according to individual circumstances. If intervention is chosen, a nephron-sparing approach should be prioritized when technically and oncologically feasible; RN may be considered when PN is not technically or oncologically feasible. Future advances in radiomics, AI, and molecular biomarkers may provide new tools for preoperative assessment of biological aggressiveness. Multidisciplinary collaboration, longitudinal follow-up, and integrated consideration of life expectancy, comorbidities, kidney function, and treatment preferences remain the foundations of clinical decision-making.

## Figures and Tables

**Figure 1:**
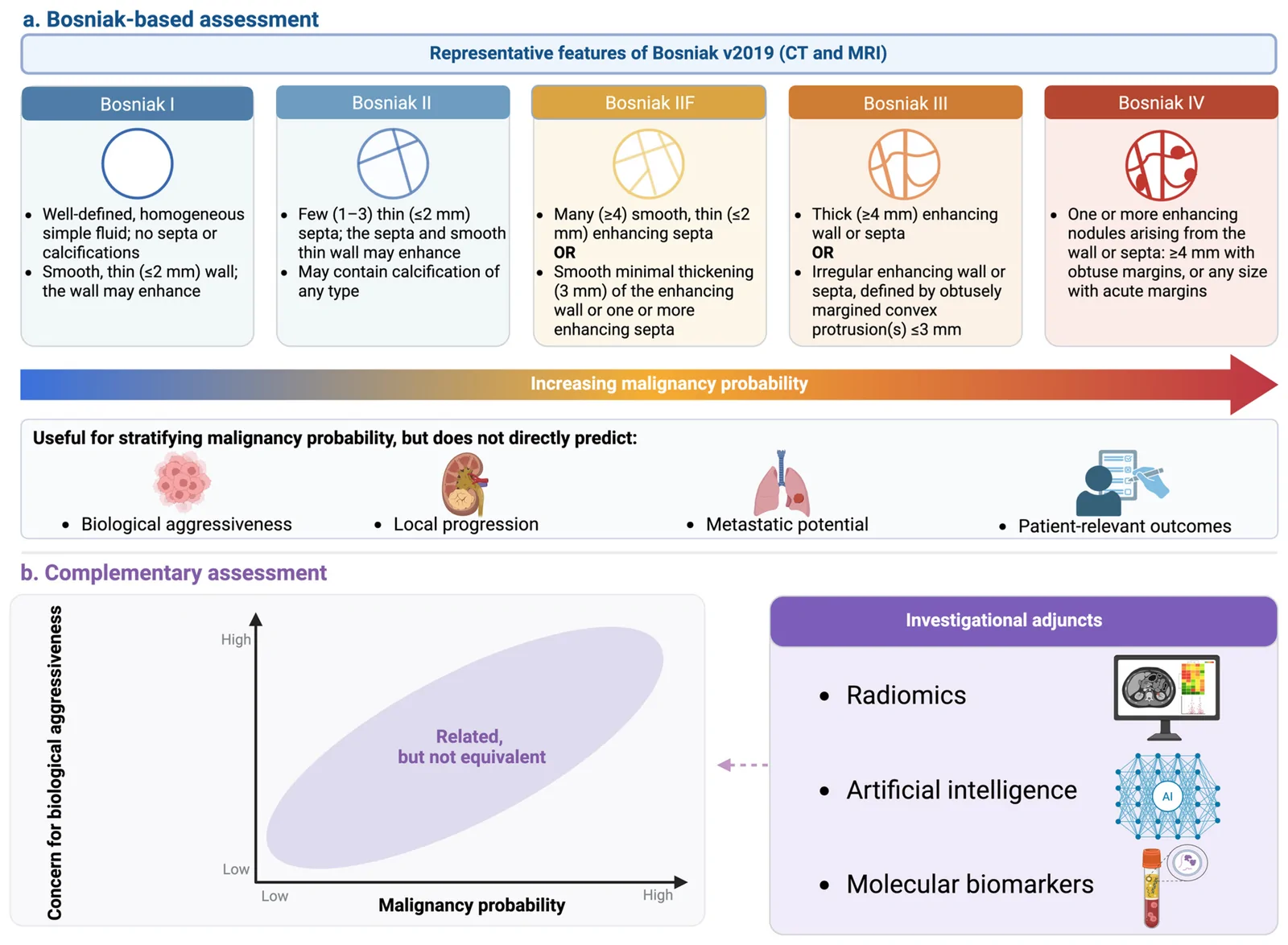
Beyond malignancy probability: a conceptual framework for the assessment of cystic renal masses. (**a**) Representative CT- and MRI-based morphologic features of the Bosniak classification version 2019, which stratifies the probability of malignancy but does not directly predict biological aggressiveness or patient-relevant outcomes. (**b**) Complementary assessment considers malignancy probability alongside concern for biological aggressiveness. The shaded field is schematic and illustrates that these dimensions are related but not equivalent; radiomics, artificial intelligence, and molecular biomarkers are presented as investigational adjuncts. Abbreviations: CT, computed tomography; MRI, magnetic resonance imaging. Created in BioRender. Qu, L. (2026) https://BioRender.com/s9wxypb (accessed on 21 September 2026).

**Figure 2:**
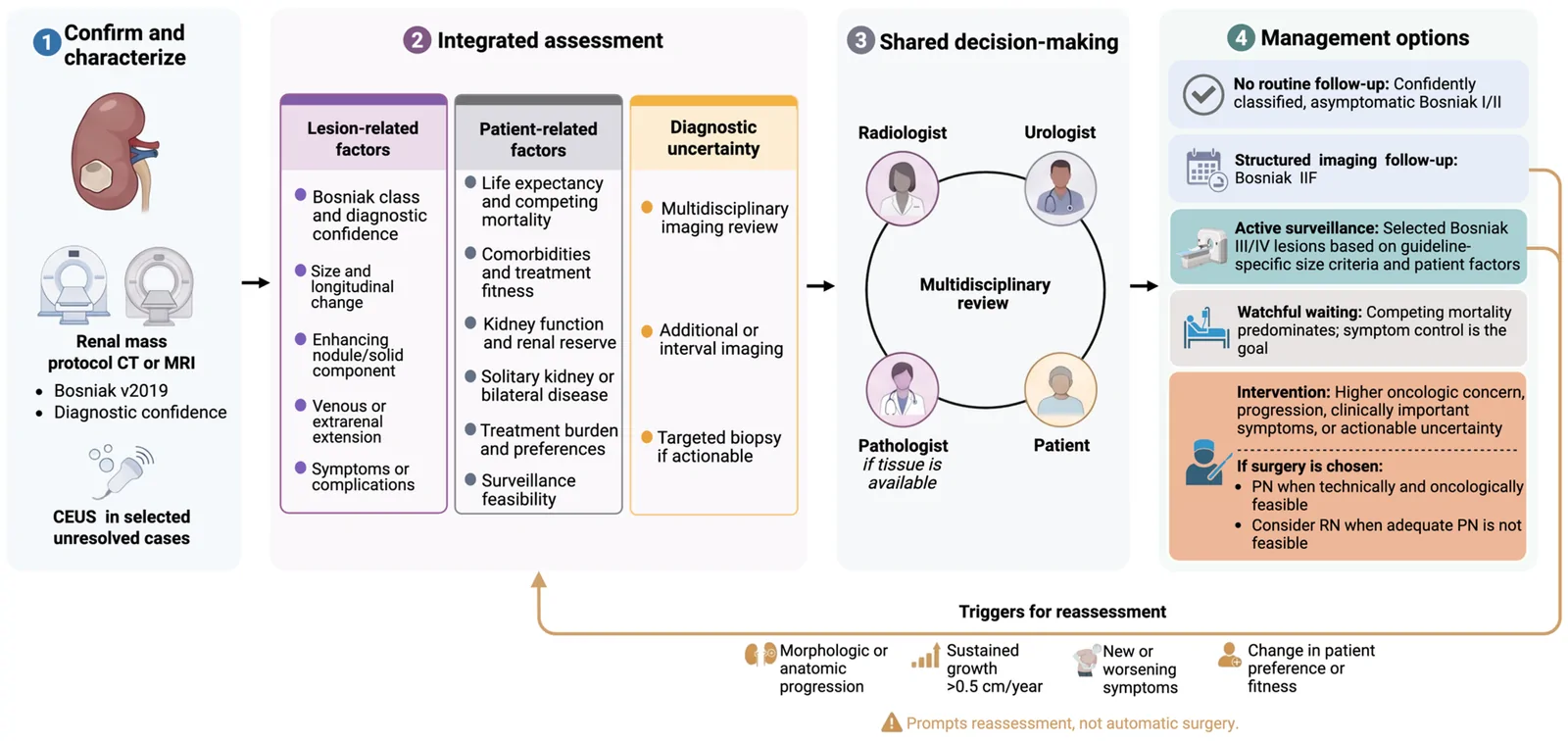
A risk-adapted and patient-centered framework for the management of cystic renal masses. Imaging findings, lesion- and patient-related factors, and diagnostic uncertainty are integrated through multidisciplinary review and shared decision-making. Changes during follow-up prompt reassessment rather than automatic surgery. Abbreviations: CEUS, contrast-enhanced ultrasound; CT, computed tomography; MRI, magnetic resonance imaging; PN, partial nephrectomy; RN, radical nephrectomy. Created in BioRender. Qu, L. (2026) https://BioRender.com/2mk2ro6 (accessed on 21 September 2026).

**Table 1:** Selected histologic entities relevant to the interpretation of cystic renal masses.

Histologic Entity	Key Histologic Features	Implications for Diagnosis and Evidence Interpretation	References
Adult cystic nephroma within the MEST spectrum	Predominantly cystic, with epithelial-lined spaces separated by spindle-cell stroma, often showing ovarian-type features.	May mimic a malignant cystic mass on imaging; definitive distinction relies on histologic examination.	[20,21]
MCRNLMP	Multiple cysts lined by low-grade clear cells, with small nonexpansile clusters of similar cells in fibrous septa; no expansile solid nodules or tumor necrosis.	Adequate tissue sampling is central to distinction from cystic ccRCC; shared chromosome 3p deletion and VHL alterations are not sufficient for this distinction.	[28,29,30]
ccRCC with cystic change	Cystic architecture accompanied by expansile nests or nodules of clear cells within septa or solid areas.	Favorable outcomes reported in selected low-grade cystic cohorts should be interpreted in the context of histologic grade and stage rather than generalized to all ccRCCs with cystic change.	[29,31]
pRCC with cystic features	Papillary or tubular architecture with variable cystic change; some tumors show a distinct microcystic growth pattern.	Imaging-defined cystic appearance is not equivalent to microscopic microcystic architecture; associations between the latter and adverse outcomes do not establish an imaging-based risk marker.	[33,34]
Tubulocystic RCC	Pure tubulocystic architecture comprising variably sized tubules and cysts lined by flattened, cuboidal, or hobnail cells with eosinophilic cytoplasm.	Outcome data from strictly defined pure tumors should not be extrapolated to other RCC subtypes with overlapping tubulocystic morphology.	[35]

Abbreviations: MEST, mixed epithelial and stromal tumor; MCRNLMP, multilocular cystic renal neoplasm of low malignant potential; ccRCC, clear cell renal cell carcinoma; 3p, short arm of chromosome 3; VHL, von Hippel–Lindau tumor suppressor gene; pRCC, papillary renal cell carcinoma; RCC, renal cell carcinoma.

**Table 2:** Evidence basis and clinical applicability of diagnostic tools and management measures discussed in this Perspective.

Evidence Category	Tool, Factor, or Measure	Clinical Role and Scope of Supporting Evidence	References
Established assessment methods	CECT/MRI and adjunctive CEUS	CECT and MRI characterize lesion morphology, enhancement, and anatomic extent; CEUS provides additional assessment of enhancement in the wall, septa, and nodules.	[1,3,40,41,42,43]
	Bosniak v2019 malignancy risk stratification	Estimates malignancy probability using imaging features of the cyst wall, septa, and enhancing nodules.	[38,39,55]
Guideline-supported measures	Selection of AS or intervention	Decisions incorporate Bosniak category, lesion size, and patient factors. For Bosniak IV lesions measuring 2–4 cm, CUA allows AS or surgery, whereas NICE favors surgery in suitable patients.	[3,66]
	Selection of PN or RN in surgical patients	For Bosniak III/IV CRMs requiring surgery, CUA favors PN over RN when technically and oncologically feasible.	[3,52,68]
Clinical study evidence	Intraoperative localization, tissue identification, and resection: ultrasonography, NIRF, and enucleation/enucleoresection	Intraoperative ultrasonography assists lesion localization, while NIRF helps distinguish exposed tumor from normal renal parenchyma; supporting studies primarily involve general renal tumor cohorts. Enucleation techniques also have application and outcome data from cystic RCC cohorts.	[70,71,72,73]
	Intraoperative frozen-section assessment of the tumor bed	A general PN cohort evaluated the relationship between tumor-bed frozen sections and final specimen margin status. Tumor-bed frozen sections cannot replace permanent-section assessment of the resected specimen.	[79]
Investigational models/markers	CT-based radiomics and deep learning for lesion delineation and benign–malignant classification	CT image analysis assists benign–malignant differentiation; some systems also support lesion delineation. Miskin’s CT texture model underwent internal cross-validation. Huang’s radiomics model and He’s deep-learning classifier achieved external validation AUCs of 0.86 and 0.998, respectively.	[44,45,46]
	Combined CT and clinical models for distinguishing indolent from aggressive lesions	Combines CT-derived malignancy probability with age, sex, body mass index, lesion location, and size to distinguish study-defined indolent and aggressive CRMs. Kang’s XGBoost model achieved AUCs of 0.92 in internal validation and 0.80 in the first external test cohort.	[47]
	Preoperative application of molecular markers	Tissue studies of VHL alterations and chromosome 3p loss support a molecular relationship between MCRNLMP and ccRCC; their use for preoperative risk assessment remains investigational.	[29,30]
Expert opinion	Preservation of cyst integrity, rupture management, and defect reconstruction: overlying fat preservation, a resection line approximately 0.5 cm from the cyst wall, suction/selective irrigation, and fat pad packing	Suction of spilled tumor tissue is supported by Delphi consensus; irrigation did not achieve consensus. Overlying fat preservation and the resection distance derive from single-center technical experience. Fat pad packing is a proposed adaptation of general PN reconstruction for CRMs.	[76,77,78]

Abbreviations: CECT, contrast-enhanced computed tomography; MRI, magnetic resonance imaging; CEUS, contrast-enhanced ultrasound; AS, active surveillance; CUA, Canadian Urological Association; NICE, National Institute for Health and Care Excellence; PN, partial nephrectomy; RN, radical nephrectomy; CRM, cystic renal mass; NIRF, near-infrared fluorescence; RCC, renal cell carcinoma; CT, computed tomography; AUC, area under the receiver operating characteristic curve; XGBoost, extreme gradient boosting; MCRNLMP, multilocular cystic renal neoplasm of low malignant potential; ccRCC, clear cell renal cell carcinoma.

## Data Availability

No new data were created or analyzed in this study. Data sharing is not applicable to this article.

## Abbreviations

The following abbreviations are used in this manuscript:

ACD-RCC	Acquired cystic disease-associated renal cell carcinoma
ACKD	Acquired cystic kidney disease
ACN	Adult cystic nephroma
ADPKD	Autosomal dominant polycystic kidney disease
AI	Artificial intelligence
AS	Active surveillance
AUC	Area under the receiver operating characteristic curve
ccRCC	Clear cell renal cell carcinoma
CD	Calyceal diverticulum
CECT	Contrast-enhanced computed tomography
CEUS	Contrast-enhanced ultrasound
CI	Confidence interval
CRM	Cystic renal mass
CT	Computed tomography
CUA	Canadian Urological Association
ESRD	End-stage renal disease
MCRNLMP	Multilocular cystic renal neoplasm of low malignant potential
MEST	Mixed epithelial and stromal tumor
MRI	Magnetic resonance imaging
NICE	National Institute for Health and Care Excellence
NIRF	Near-infrared fluorescence
PN	Partial nephrectomy
pRCC	Papillary renal cell carcinoma
RAPN	Robot-assisted partial nephrectomy
RCC	Renal cell carcinoma
RN	Radical nephrectomy
SRC	Simple renal cyst
WHO	World Health Organization
XGBoost	Extreme gradient boosting
